# A Practical Treatise on Medical Diagnosis

**Published:** 1901-01

**Authors:** 


A Practical Treatise on Medical Diagnosis. For the use of students and practitioners. By John H. Musser, M. D., Professor of Clinical Medicine, University of Pennsylvania, Philadelphia. New (fourth) edition, thoroughly revised. In one octavo volume of 1104 pages, with 250 engravings and 49 full-page colored plates. Philadelphia and New York: Lea Brothers & Co. 1900. [Price, cloth, $6.00 net; leather, $7.00 net; half morocco, $7.50 net.]

The fourth edition of Musser's Medical Diagnosis is demanded although its predecessor was published less than a year ago. The rapid exhaustion of the third edition is a gratifying evidence that the author's efforts have been duly appreciated, and that more attention is being paid to the art of making careful diagnoses.

Succesful treatment, the aim of every practitioner, can follow only an accurate and complete diagnosis, and this in turn demands the use of every known method of investigating symptoms and conditions, however complicated or obscure. To no more trustworthy, authoritative, modern or comprehensive book can the practitioner refer in times of anxiety and doubt than the one under consideration.

The work is divided into two parts; Part I. dealing with general diagnosis and Part II. with special diagnosis.

Under Part I. several chapters are devoted to the study of data obtained by inquiry, the careful investigation of the onset, and course of the disease in question, the early history, family history, age, sex, occupation and habits and environment of the patient, giving the physician a bird's-eye view of the case before he undertakes to obtain data by observation. In the chapters allotted to the examination of the patient, the facts obtained are carefully classified and aids thrown out to the reader to help him to correctly interpret their meaning. A work which is constantly passing from edition to edition is able to keep abreast of the times and enables the author to keep his work carefully revised to the latest date; such revision has been accomplished in the present edition as Koplik's latest paper on the measles spots in the buccal cavity, on page 260, will testify. The latest investigations on the normal and pathological condition of the blood have been incorporated, and the blood plates are beautifully and accurately executed.

The special diagnosis of the various organs of the body follows, the author starting with the nose and larynx, followed by chapters on the lungs and pleura, heart, bloodvessels, mediastinum, mouth, fauces, pharynx, esophagus, stomach, intestines, liver, spleen, pancreas, kidneys and diseases of the nervous system.

If any of the chapters on the special organs were to be more highly lauded than the others it would be that of the lungs and pleura. Here the author displays his greatest generalship in marshaling the many intricate and troublesome facts concerning the respiratory tract, and with an ease and scientific grace that calls forth admiration. The plates with the platting of the relations between the various organs of the chest and abdomen are great helps to the enquiring physician, and cannot help but give what he seeks—information. The chapter on the disease of the nervous system is well written, but rather scant for such an important subject and the author has abridged and crowded many facts into rather narrow confines.

Instruments of precision, the topography of disease and every accepted method of clinical and bedside investigation are so clearly and fully described that Musser's Diagnosis must take its place among the very best works of its kind and therefore appeals to the student and to the practitioner as a never failing guide and consultant.

The illustrations have been revised as thoroughly as the text, and beside 250 engravings, the book contains no fewer than forty-nine full-page colored plates. The best of the bookmakers' art has been expended in bringing this book before the medical world.

